# Cultivating Intergenerational Exchange: An Age-Friendly University Model for Strategic Partnership

**DOI:** 10.1093/geroni/igaa057.1733

**Published:** 2020-12-16

**Authors:** Elizabeth Bergman, Mary Ann Erickson, Jessica Valdez Taves

**Affiliations:** Ithaca College, Ithaca, New York, United States

## Abstract

Having recently celebrated its 20th anniversary, the Ithaca College/Longview Partnership is an enduring part of the Ithaca College experience. Longview is a senior living community located just across the street from campus on land donated by the college. A formal partnership has been in place since 1999, which provides a structure of support for various shared services and mutual access to educational and social opportunities for Ithaca College students, faculty, and staff, and Longview residents and staff. This presentation will include a brief overview of the history of the IC/Longview partnership, describe the infrastructure necessary for a successful partnership, and highlight some recent specific, high-impact programs that have made active engagement between Ithaca College and the Longview community possible.

